# SIMPLE 3.0. Stream single-particle cryo-EM analysis in real time

**DOI:** 10.1016/j.yjsbx.2020.100040

**Published:** 2020-11-07

**Authors:** Joseph Caesar, Cyril F. Reboul, Chiara Machello, Simon Kiesewetter, Molly L. Tang, Justin C. Deme, Steven Johnson, Dominika Elmlund, Susan M. Lea, Hans Elmlund

**Affiliations:** a Sir William Dunn School of Pathology, University of Oxford, Oxford, UK; b Central Oxford Structural Microscopy and Imaging Centre, University of Oxford, Oxford UK; c Department of Biochemistry and Molecular Biology, Biomedicine Discovery Institute, Monash University, Melbourne, Victoria, Australia; d Australian Research Council Centre of Excellence in Advanced Molecular Imaging, Monash University, Melbourne, Victoria, Australia

**Keywords:** Single-particle, Cryo-EM, Real-time, Stream image processing

## Abstract

We here introduce the third major release of the SIMPLE (Single-particle IMage Processing Linux Engine) open-source software package for analysis of cryogenic transmission electron microscopy (cryo-EM) movies of single-particles (Single-Particle Analysis, SPA). Development of SIMPLE 3.0 has been focused on real-time data processing using minimal CPU computing resources to allow easy and cost-efficient scaling of processing as data rates escalate. Our stream SPA tool implements the steps of anisotropic motion correction and CTF estimation, rapid template-based particle identification and 2D clustering with automatic class rejection. SIMPLE 3.0 additionally features an easy-to-use web-based graphical user interface (GUI) that can be run on any device (workstation, laptop, tablet or phone) and supports a remote multi-user environment over the network. The new project-based execution model automatically records the executed workflow and represents it as a flow diagram in the GUI. This facilitates meta-data handling and greatly simplifies usage. Using SIMPLE 3.0, it is possible to automatically obtain a clean SP data set amenable to high-resolution 3D reconstruction directly upon completion of the data acquisition, without the need for extensive image processing post collection. Only minimal standard CPU computing resources are required to keep up with a rate of ∼300 Gatan K3 direct electron detector movies per hour. SIMPLE 3.0 is available for download from simplecryoem.com.

## Graphical abstract

**Figure:**
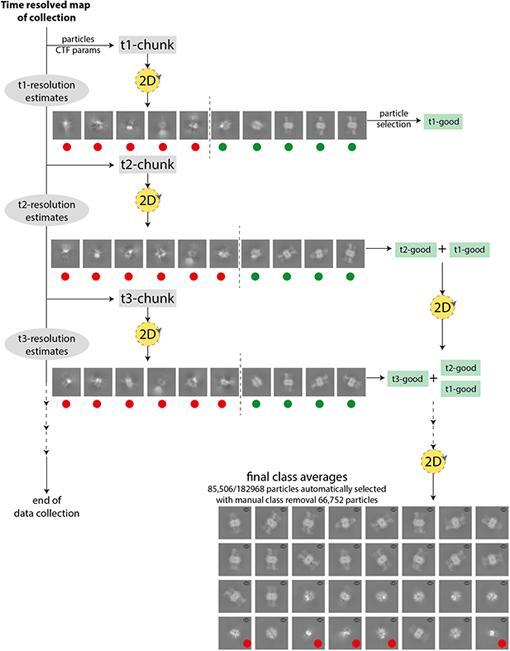

## 1 Introduction

**Fig. 1:**
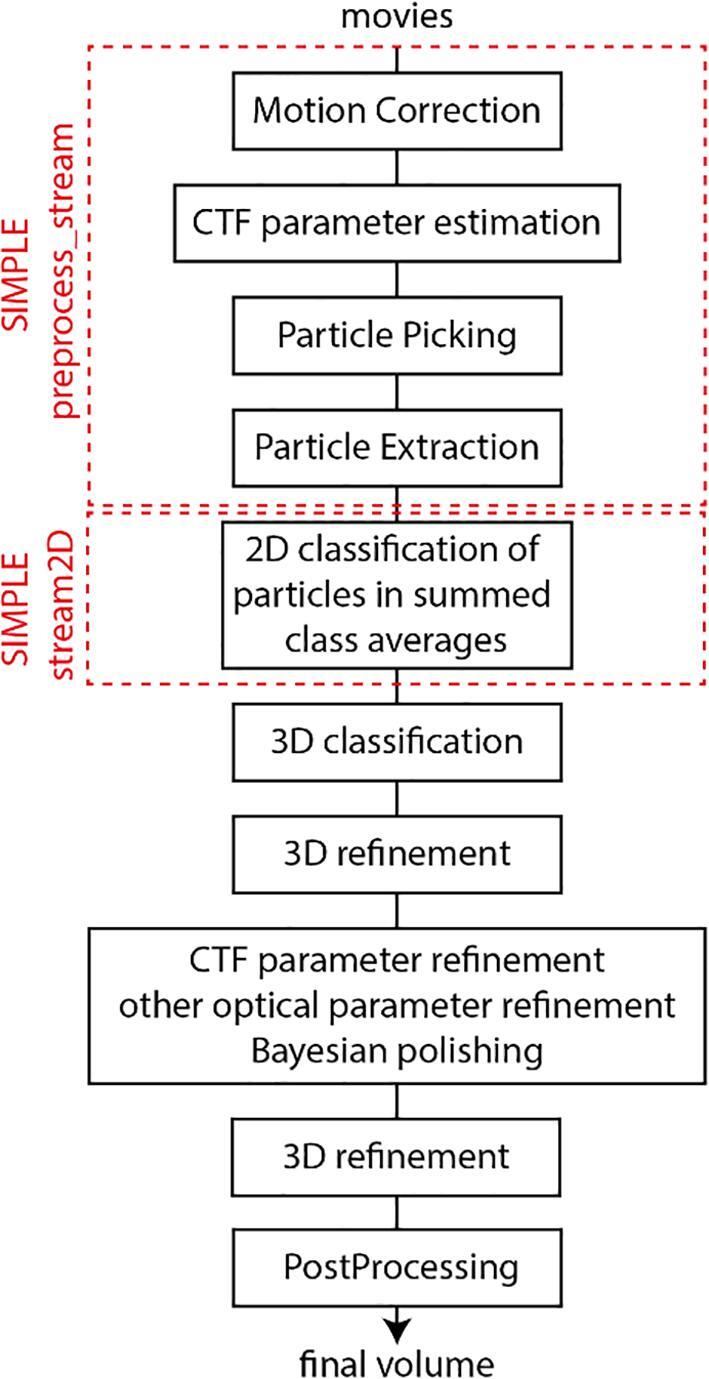
Schematic overview of steps in SPA.

Over the last few years, the emergence of a new generation of electron microscopes, with new direct electron detectors (Grigorieff, 2013, McMullan et al., 2009, McMullan et al., 2014) and improved algorithms for image analysis (Grant et al., 2018, Ludtke, 2016, Punjani et al., 2017, Reboul et al., 2016, Reboul et al., 2018a, Reboul et al., 2018b, Yang et al., 2012, Zivanov et al., 2018) have enabled routine determination of the 3D structure of biological molecules by cryo-EM and Single-Particle Analysis (SPA). It is now possible to determine near-atomic resolution (<4 Å) structures of biomolecules below 100 kDa in mass in favorable cases (Khoshouei et al., 2017) and solve sub-2 Å-resolution structures of larger macromolecules (Bartesaghi et al., 2018). Although possible, *de novo* structure determination by SPA is still challenging and generally requires many cycles of sample optimization following collection and data analysis. Initial analysis of SP data consists of a series of established processes (Fig. 1).

Several software packages provide different algorithmic solutions for all or some of these processes (Grant et al., 2018, Hohn et al., 2007, Punjani et al., 2017, Reboul et al., 2018b, Scheres, 2012, Tang et al., 2007). Traditionally, SPA was performed post collection, with manual intervention between the processing steps, limiting the use of downstream information to rapidly inform decisions about sample optimization and microscope use. The production of 2D class averages to visualize and analyze the statistics of signal-enhanced averages of particles with similar projection direction is key to determining data quality (Reboul et al., 2016, Scheres et al., 2005, van Heel, 1984, Yang et al., 2012). It is therefore desirable to have this analysis available as early as possible during the collection, requiring automation and acceleration of the operations involved to allow near real-time analysis. Data collection rates are rapidly increasing with new generations of detectors producing up to 10,000 movies per day (often 2-5 × 10^6^ particles/day). This is escalating the computational demands but opens the possibility of rapid, definitive assessment of samples at the 2D level within the first hour of data collection. Several developers are working to provide solutions for this real-time problem (Gomez-Blanco et al., 2018, Maluenda et al., 2019, Tegunov and Cramer, 2019, Wagner and Raunser, 2020). We here describe SIMPLE 3.0, which we have developed to managed data flow in the Central Oxford Structural Molecular Imaging Centre over the last two years. SIMPLE 3.0 runs on relatively cheap and scalable CPU resources thus allowing use of existing computer resources to support real-time processing of SP data (Table 1, Results below). In contrast to developments that provide data organization tools and interfaces to other software, such as Scipion (Gomez-Blanco et al., 2018, Maluenda et al., 2019) and Focus (Biyani et al., 2017), our SIMPLE 3.0 suite consists of a collection of novel image processing algorithms that have been tailored and optimized for use in a stream scenario.

**Table 1:** Typical CPU resources required to keep up with data generated by the given detectors at the rate shown. All benchmarks were performed on machines with AMD EPYC 7551P processors, 192 GB RAM and an SSD backed BeeGFS filesystem. These minimal resources can easily be housed within a single processing machine using modern CPU hardware.

Detector	Movie Dimensions	Movie Frames	Movies/hour	CPU Threads
K2	3838 × 3710	32	100	16
K3(super-resolution)	11520 × 8184	40	300	88

## 2 Results

The SIMPLE 3.0 suite currently contains more than 50 individual programs and more than 20 distributed workflows; here we will focus on implementation of the streaming workflows. We aim to provide the highest possible performance and efficiency on any CPU hardware, from supercomputers to everyday workstations or even laptops. Therefore, distributed workflows implement larger tasks that can be run in a cluster environment or on a high-powered workstation, whereas programs implementing smaller tasks may be run on any computer. Key to the use of SIMPLE 3.0 to stream data within our facility has been the development of a graphical user interface for all program interactions. Easy routes to export data post selection based on 2D analysis allow users free choice in software used for downstream 3D operations; e.g. stay within the SIMPLE 3.0 package or use alternates such as RELION 3.1 (Zivanov et al., 2020) or cryoSPARC (Punjani et al., 2017) among others. The source code, as well as introductory, tutorial, installation, usage, reference and developer information are available at simplecryoem.com. SIMPLE 3.0 is free software distributed under version 3 of the GNU general public license and includes a CMake build environment to simplify compilation and installation. Various external libraries are either bundled for convenience or are freely available, whilst the SIMPLE suite itself is compiled using GCC. The public git repository is available at https://github.com/hael/SIMPLE.git and the 3.0.0 release is available at https://github.com/hael/SIMPLE/releases/tag/v3.0.0.

### 2.1 Graphical user interface

**Fig. 2:**
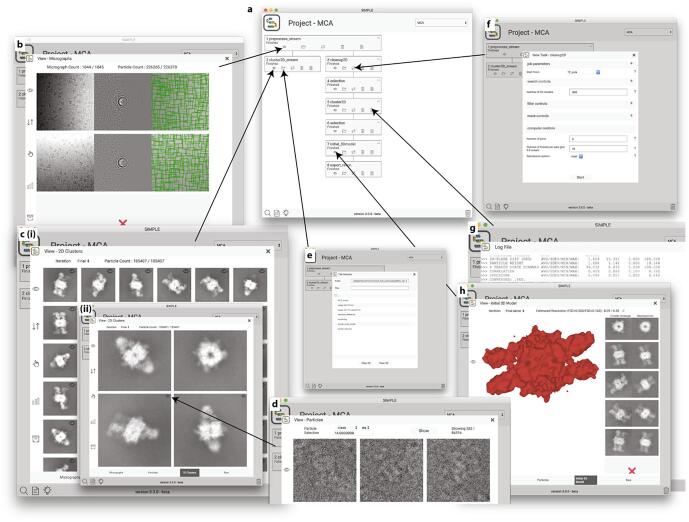
Graphical User Interface (GUI). (a) Project window with workflow graph outlining the executed processes. Each box represents a process with the execution directory as heading and a process indicator (running, finished, failed) just below. The clickable eye icon in the lower left corner of each box links to (b) viewable outputs. In this example, the (b) panel shows micrograph (left), background subtracted power spectrum/fitted CTF model (middle) and picked particle coordinates (right) generated by 1_preprocess_stream. (c(i)) Following stream 2D analysis (process #2, executed after #1 stream pre-processing), the viewer links to the class averages produced. (c(ii)) The class averages can be closely inspected and link to (d) a particle viewer via the eye icon in the upper right corner of each class average, allowing visualization of the particles associated with each class and inspection of their associated statistics. (e) The folder icon in each box allows inspection of the output files produced in the execution directory. Outputs that can be rendered on screen link to viewers. (f) The process icon (cyclic double arrow) link to the task control window, where input parameters are arranged in dropdown menus according to their categorization. Only dropdown menus with required inputs are expanded by default. In this example, the categories are job parameters, search controls that modify optimization behavior, filter controls that modify Fourier filtering behavior, mask controls and computer controls used to change how the task is executed, *i.e.* number of threads etc. (g) The text file icon allows inspection of the log file, to which all SIMPLE 3.0 subprocesses concatenate their output. The log file is used to report subprocess exceptions and should be inspected when the process indicator is in the “failed” state. (h) When 3D volumes are available they can be visualized and the volume viewer supports 3D rendering over remote connection. Shown here is the output from 7_initial_3Dmodel, which in addition to the initial 3D reconstruction shows the class averages used and the associated re-projections of the volume for validation.

We have developed a GUI which organizes executed processes by project and represents them in a workflow diagram (see Fig. 2a). This provides improved organization and makes it possible to restart or export a project at any point in a workflow. Results can be inspected by viewers that have been optimized for each task. The GUI front end is written in HTML, CSS and JavaScript, which has the advantage that it can be run on any device with a modern web browser (workstation, laptop, tablet or phone) and across platforms. Furthermore, it can handle 2D and 3D image rendering across remote http/https connections to clusters using server-side image conversion to compressed JPEG format and automatic down sampling of volumes coupled with conversion to BinaryCIF by the DensityServer data delivery service (Sehnal et al., 2017). The GUI backend has an integral HTTP server and may be run in standalone mode, running transiently for the current user, or as a permanent service in multi-user mode, allowing multiple users with different login credentials to utilize the server. In a cluster environment, the GUI must be run on a machine which can submit jobs to the available computing resources and made accessible to users via the HTTP protocol. Configuration examples are available in the installation instructions.

The viewers within the GUI enable inspection of log files, *i.e.* individual process text output, project files and allows visualization of 2D and 3D MRC files, including individual micrographs, power spectra, theoretical CTF model, picking coordinates, extracted particles, 2D class averages and 3D volumes (using LiteMol (Sehnal et al., 2020))—all within a web browser window (see Fig. 2). The viewers allow selection/deselection to be made at any point. Selections are saved as a node in the workflow, so that different selections can be readily used for downstream processes.

For improved usability, all program/workflow names describe their functionality, e.g. *motion_correct, ctf_estimate, pick, cluster2D, initial_3Dmodel, refine3D* etc. The number of control parameters associated with a program/workflow can often be daunting and to simplify their representation, we have divided them into categories represented by drop-down menus in the GUI. Only required parameters are shown when first opening a task control window and optional parameters are accessed through expansion of the menus. A program or workflow is executed in a directory with the name *X_program_name*, where *X* is the sequential execution directory number. A project file storing metadata describing the job and its output is stored alongside the output data within this execution directory, allowing nonlinear execution paths; for example, multiple 2D clustering rounds from the same starting point with different number of clusters or execution of multiple 3D refinement jobs with different input parameter settings. The GUI allows clean-up of a cluttered workflow and removal of the associated execution directories.

In SIMPLE 3.0, we automatically generate an abstract user interface description (UI) within the back-end using Java Script Object Notation (JSON). The UI JSON file establishes a one-to-one correspondence between the command-line driven back-end of SIMPLE 3.0 and the GUI front-end. This allows the command-line descriptions to be built and organized automatically from the back-end, allowing back-end developers to modify existing functionalities and introduce new programs/workflows without worrying about outdating the GUI. The back-end developers are currently located in Melbourne, Australia and the GUI developers in Oxford, UK. Therefore, this abstraction was created to improve the communication between developers through establishing a common language when talking about specific SIMPLE functionalities. This kind of abstraction ought to be helpful also for those developing packages of packages, such as Scipion (de la Rosa-Trevín et al., 2016, Gomez-Blanco et al., 2018) or Appion (Lander et al., 2009), allowing more rapid integration when new versions of SIMPLE are released. Furthermore, it could provide a framework for integrating other functionalities than SIMPLE within the GUI in the future.

### 2.2 Motion correction

Sample motion affects direct electron detector movies in two ways: whole-frame motion (stage drift) and anisotropic (beam-induced) local motion (Brilot et al., 2012, Campbell et al., 2012). We developed an algorithm that corrects for both effects in a sequential manner through determining (1) the two-dimensional shifts that describe the isotropic (whole-frame) motions and (2) a deformation model describing the beam-induced motions. Whole-frame correction optimizes the correlations of the individual frames to an iteratively evolving reference (e.g., the integrated movie—a weighted average of all frames parameterized with respect to shifts and frame-weights). This is done through a two-step registration scheme that begins with a rapid coarse optimization step, similar to (Zivanov et al., 2018). The following refinement step uses continuous Limited-memory Broyden–Fletcher–Goldfarb–Shanno optimization with Bound constraints (L-BFGS-B) (Byrd et al., 1995) optimization with analytical gradients of the correlations. Simultaneously, individual correlation-based frame weights are derived to marginalize the influence of the flanking frames: the first typically fast-moving frames and the last frames, where radiation damage becomes pronounced (Brilot et al., 2012, Grant and Grigorieff, 2015). This approach overcomes the need for omitting a subjective set of frames and conducting time-consuming re-processing of the data post collection (see Supplementary Material for further details). To derive a deformation model and correct for local anisotropic motion, each frame, after application of the global shifts previously determined, is subdivided into evenly distributed patches, similar to (Zheng et al., 2017). Each patch (typical size >800 pixels) is independently subjected to an optimization strategy similar to that used for whole frame motion correction. Finally, a spatiotemporal deformation model is obtained by least squares fitting of all the shifts of the frame patches to a 3D polynomial function of the third order over the exposed area (space) and throughout the exposure (time), as done in *Motioncor2* (Zheng et al., 2017). This smooth mapping thus associates each frame pixel with a set of shifts that are used to correct for the local motion and sum the interpolated and individually weighted frames to generate the final dose-weighted micrograph (Grant and Grigorieff, 2015). In both the stage drift and beam-induced motion correction phases, the shifts can be associated with a discontinuous or jittery trajectory that do not realistically represent the motion. This may be due to the low contrast in movies collected close to focus or due to the weaker signal present in patches, which can be an order of magnitude smaller than the entire exposed area. Therefore, we have implemented two mechanisms to smooth the shift trajectories: (1) To mitigate early over-fitting, the resolution limit used for alignment is iteratively updated from an initial 8 Å to a final 5 Å. Additionally, the influence of the higher and noisier resolution shells is dampened by the use of B-factor (default B-factor value is 50 Å^−2^). (2) To alleviate the emergence of significantly discontinuous shifts, the current parameters for all frames of each patch and at each iteration are interpolated using 1D polynomial functions (third order, along the x-/y-dimensions as a function of time) during the coarse optimization step. In SIMPLE 3.0, smoothing of the shift trajectories is an integral part of the optimization process and not solely regarded as a corrective procedure prior to the generation of the integrated movie. We performed comparative 3D refinements of three publicly available datasets that have previously yielded near-atomic resolution maps to validate our anisotropic motion correction strategy. In each case, when our motion correction employed the proposed isotropic and anisotropic corrections vs. isotropic only we obtained improved resolution, as determined by the gold-standard Fourier shell correlation (FSC) criterion 0.143 (Supplementary Fig. 1). The GUI displays the output of motion correction as a set of thumbnails with adjustable contrast, zoom and size. Statistics may be viewed and plotted and motion tracks for each micrograph displayed. Visual selection of good/bad micrographs can be performed and saved for downstream processing. Per-micrograph star files containing the polynomial model parameters are written by default to provide compatibility with RELION and provide support for Bayesian particle polishing (Zivanov et al., 2019).

### 2.3 Estimation of defocus and astigmatism

In SIMPLE 3.0, we adopt the CTF model put forward by (Fernando and Fuller, 2007) where the modulation of image formation in the weak-phase approximation is expressed by a two-dimensional function of the spatial frequency vector ***g*** (of length *N*) and depends on the electron wavelength λ, the objective lens defocus Δ*f*, the spherical aberration constant *C_s_*, the contrast term *A* and optionally the phase shift introduced by the Volta phase plate Δ*ϕ*:

$CTF\left(g\right)=-\sin\left[\pi \lambda g^{2}\left(\Delta f-\frac{1}{2}\lambda^{2}g^{2}C_{s}\right)+\Delta \phi +A\right]$, (1)where the defocus (subject to the angle of astigmatism α*_a_*) and the contrast term *A* are defined as:

$\Delta f=\frac{1}{2}\left[\Delta f_{x}+\Delta f_{y}+\left(\Delta f_{x}-\Delta f_{y}\right)\cos\left(2\left[\alpha \left(g\right)-\alpha_{a}\right]\right)\right]$, (2)

$A=\tan^{-1}\left(w/\sqrt{1-w^{2}}\right)$ (3)

with Δ*f_x/y_* the objective lens defocus along the image *x/y* normal directions, α(***g***) the angle between ***g*** and the *x*-axis and *w* the relative amplitude contrast. This CTF model is used in the popular CTFFIND4 program (Rohou and Grigorieff, 2015), from which we also adopt the methodology and scoring function to recover robust global micrograph estimates of the defoci Δ*f_x/y_*, astigmatism α_a_ and, when appropriate, the phase shift Δϕ (otherwise set to 0). Briefly, the per-micrograph CTF parameters are estimated by maximizing the cross-correlation between the background-subtracted micrograph power spectrum and the theoretical CTF model. The motion-corrected micrograph is evenly partitioned into overlapping square tiles (typically 512 × 512 pixels, 50% overlap) from which the respective spectra are calculated and averaged prior to background subtraction and central cross dampening to yield a final 2D spectrum *F(****g****)*. This spectrum is matched against the theoretical spectrum expressed by equation 1, using the correlation *cc* as a scoring function, calculated within a resolution range of 30 to 5 Å (Mindell and Grigorieff, 2003, Rohou and Grigorieff, 2015)

$\mathrm{cc}=\frac{\sum_{g}F\left(g\right)\left|CTF\left(g\right)\right|}{\sqrt{\sum_{g}{F\left(g\right)}^{2}\sum_{g}{CTF\left(g\right)}^{2}}}$ (4)

Maximization of the correlation between the experimental and theoretical spectra is done in three steps:

1) The astigmatism is first ignored and the 2D spectrum rotationally averaged. Correlating this 1D experimental spectrum with the CTF model (equation 1; α*_a_* = 0 and Δ*f_x_ =* Δ*f_y ;_* where relevant Δϕ *=* $\frac{\pi }{2}$) with uniformly sampled defocus values (typically within 0.3–5.0 μm) provides a non-astigmatic estimate of the defocus.
2) Next, the angle of astigmatism *α_a_* is obtained using non-linear stochastic maximization (Differential Evolution (Storn and Price, 1997)) of the correlation versus the 2D spectrum while the values of Δ*f_x/y_* are refined with restraints (+/− 0.1 μm) to favor modest astigmatism. Optionally, the Volta phase plate-induced phase shift is an additional degree of freedom in this optimization step.
3) Finally, defoci are optimized using the scoring function *f* (equation 5) consisting of *cc* and a penalty term *f_pen_* aimed at favoring solutions with modest astigmatism, consistent with the previous step and following (Rohou and Grigorieff, 2015). The defocus tolerance ΔΔ*f_tol_* is set to 0.05 μm by default. The continuous optimization is performed with analytical gradients (with α*_a_* and Δϕ are kept constant) and the L-BFGS-B optimizer (see Supplementary Material for further details).

(5) $$f=cc+f_{\mathrm{pen}}\;with\;f_{\mathrm{pen}}=-\frac{1}{2N}{\left(\frac{\Delta f_{x}-\Delta f_{y}}{{\Delta \Delta f}_{\mathrm{tol}}}\right)}^{2}$$

To estimate the quality of the fit of the parameters and assist in selecting ‘good’ micrographs for further processing we report the score *f* and the highest resolution at which a reasonable fit is achieved (*CC_fit_* = 0.75 as in CTFFIND 4 (Rohou and Grigorieff, 2015)).

The use of per-micrograph CTF parameters in subsequent single-particle 3D orientation refinement has efficiently aided the determination of near-atomic resolution density maps of numerous macromolecules. However, per-micrograph estimates do not account for artefacts inherent to the data collection such as varying specimen height that is likely to affect the accuracy of the CTF parameters at the single-particle level. Therefore, approaches have been developed to estimate per-particle local CTF parameters. These methods are typically based on the fitting of the weighted average spectra of single particles in the vicinity of one another (Su, 2019, Zhang, 2016). In a typical workflow, per-micrograph CTF parameters are preserved during 2D classification and 3D refinement while determination of per-particle parameters is delegated to a subsequent refinement step, employing a set of neighboring single-particle images (Punjani et al., 2017, Zivanov et al., 2018). In contrast, we implemented a patch-based CTF fitting approach related to (Tegunov and Cramer, 2019) that associates local defocus values to any arbitrarily located single-particle on the micrograph. Our algorithm is not part of the 3D refinement step but estimates an anisotropic CTF model from the integrated movie and has been optimized to suit online processing. To derive anisotropic CTF parameters, the algorithm follows the steps:

1) A square grid is built covering the exposed area with points spaced by 512 pixels. Each grid point is associated with a set of neighboring tiles, a subset of the ones generated for per-micrograph CTF estimation (see above), and a weighted average of the corresponding power spectra calculated. Our CTF ‘patch’ thus refers to a distance-weighted average of the tiles spectra assigned to each grid point. The distance weights *w_ij_* of tile *j* to grid point *i* distant by *d_ij_* are computed as

$w_{\mathrm{ij}}=\frac{e^{-{\frac{1}{2}d}_{\mathrm{ij}}^{2}}}{\sum_{k}^{N_{t}}e^{-{\frac{1}{2}d}_{\mathrm{ik}}^{2}}}$, (6)

with *N_t_* the number of neighboring tiles. In practice, a patch consists of *N_t_* = 32 radially sampled tiles that contribute more than 1% to the spectrum. Notably, our ‘patch’ definition differs from that adopted in motion correction methods where patches are generally treated independently of each other and are often separated by over 800 pixels.

2) For each grid point and associated average spectrum, CTF parameters are estimated using our above-described continuous optimization strategy, with the angle of astigmatism kept constant.
3) A global defocus variation model is fitted to both Δ*f_x_* and Δ*f_y_* using a 2-dimensional polynomial function of the third order. An 4000 × 4000 pixels micrograph would contain 49 grid points, which provides fine enough sampling for accurate spatial fitting of the polynomial function. Each pixel of the micrograph maps to a set of smooth local CTF parameters, which concludes our anisotropic CTF fitting.

Our anisotropic CTF estimation method associates each single-particle extracted from the micrograph with local CTF parameters that will immediately benefit subsequent 2D and 3D analyses. The SIMPLE 3.0 implementation of the method has been optimized for efficient real-time analysis and provides support for under-focused Volta phase-plate images and fitting of the associated phase shift. The GUI displays the output of the CTF estimation as a set of thumbnails containing a user defined selection of the micrograph, power spectrum pre and post motion correction and the power spectrum with the resulting CTF fit, all with individually adjustable contrast, zoom and size. Statistics may be viewed and plotted whilst visual selection of good/bad micrographs can be performed and saved for downstream processing.

### 2.4 Particle identification and extraction

Template-based particle picking has been part of SIMPLE since the 2.5 release (Reboul et al., 2018a) and the method has been demonstrated to work well in real-life (Kuhlen et al., 2018, Lauber et al., 2018). Our picker takes a set of class averages or re-projections of a volume and uses the fast local correlation method (Roseman, 2003) to produce one “correlation image” per reference. The correlation image used for identification of peaks is defined as the pixelwise maximum among all the correlation images. A two-dimensional version of Otsu’s algorithm (Otsu, 1979) is used to segment the final correlation image into peak and non-peak regions and create a mask for accelerating identification of peaks. Binary segmentation is applied again to identify particle positions. Aggregations and false positives are eliminated with a distance filter. When a pair of peaks are closer than a distance threshold (2.7 times the maximum particle radius by default) only the highest peak is preserved. Outlier detection based on statistical analysis of the standard deviation, average value of the power spectrum and dynamic pixel range calculated in a window around the particle positions is used to discard false positives.

The major limitation of our or any other template-based picker, when applied in a streaming scenario, is that the user needs to manually process a number of micrographs to produce template class averages. However, this can be rapidly done through a manual picking function in the GUI or, as users have found, in many cases a sufficiently good template can be provided by use of reprojections or 2D class averages from another project with a similar MW target or gaussian blob of suitable size, removing the need to manually pick the data to obtain references. However, if the user wishes to manually pick, the GUI displays the output of picking in a large window with adjustable contrast, brightness and zoom, with the option of applying a blur transformation to maximize particle contrast. Picked boxes can be displayed as squares or circles with any arbitrary size, to aid assessment of the picking quality. Upon extraction, particles are normalized exactly as in RELION 3.0 to provide forward compatibility with ML-based refinement implementations.

### 2.5 2D stream analysis

In two previous papers (Reboul et al., 2016, Reboul et al., 2018b), we have shown that our stochastic hill climbing-based algorithm for 2D analysis is a rapid and powerful tool for obtaining high-quality class averages from cryo-EM images in an unsupervised manner. A critical aspect is to be able to distinguish particles of poor quality and contaminations, which will inevitably make up a fraction of what is picked, from high-quality particles. Our tests indicate that the gold-standard resolution estimates obtained (one per class) directly reflect the quality of the particles that are members of the class. This constitutes the basis for automatic class rejection, either by a user-defined input resolution boundary or an automatically estimated one. One of the challenges is how to design a clustering algorithm that provides a time-resolved view of the data acquisition, whilst simultaneously refining a global clustering solution. The global solution is necessary for monitoring the resolution improvement in a global sense, *i.e.* as more data is collected the resolution should improve up to the point where enough data has been collected. We therefore analyze the incoming data in chunks of pre-defined size to provide a time-resolved map. A watcher waits for a sufficient number of particles meeting the quality criteria to be acquired and then launches the first 2D analysis chunk. Low-quality classes and their corresponding particles are automatically rejected based on gold-standard resolution estimates. As further chunks are acquired, both particles and classes are added to the global refinement and the number of global classes is dynamically expanded. The final result is a time-resolved map of the data collection (per-chunk class averages) in addition to a globally and iteratively refined solution. The global resolution estimates can be analyzed in context of the chunk estimates to identify the best chunks of data, *i.e.* those that gave rise to the largest global resolution improvements.

**Fig. 3:**
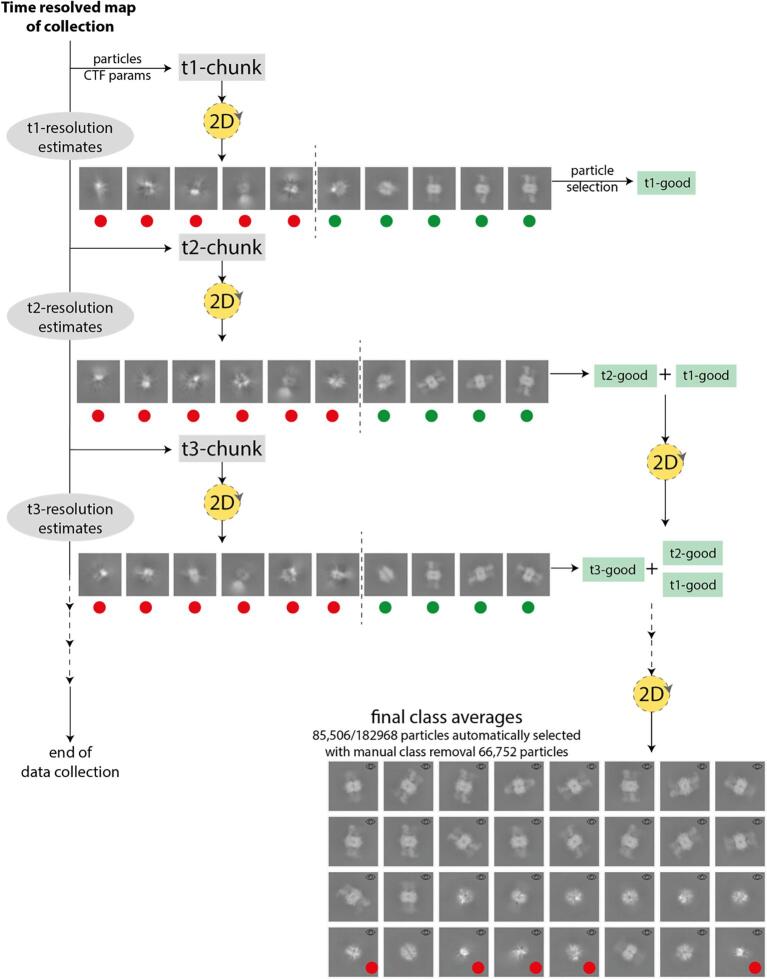
Schematic overview of 2D stream processing. Red/green dots indicated good/bad classes. In the final class averages the red dots indicate an additional 5 classes manually deselected in addition to those automatically rejected during the streaming processing. The resolution of the best class averages is estimated to 7.9 Å. (For interpretation of the references to colour in this figure legend, the reader is referred to the web version of this article.)

Fig. 3 provides a schematic overview of the proposed approach in addition to results we obtained from cryo-EM images of *Stenotrophomonas maltophilia* methylcrontonoyl-CoA carboxylase, obtained in the Oxford facility. The best classes in the global solution are resolved to better than 7.9 Å resolution, whereas the classes included from the chunks are resolved to just below 10 Å.

During stream 2D processing, classes and alignment parameters improve continuously during the acquisition. To prevent early rejection of meaningful classes (rare views, for example) we implemented an automated ‘soft’ rejection strategy, where classes of low quality are progressively rejected. The resolution estimate *res_i_* (and associated spectral index *g_i_*) of each class *i* is derived from the FRC = 0.143 criterion, with average $\mu_{g}$ and standard deviation $\sigma_{g}$. The average class correlation *cc_i_* is the average of the correlations of class average *i* to all particles belonging to the class, with $\mu_{\mathrm{cc}}$ and $\sigma_{\mathrm{cc}}$ the average and standard deviation of *cc_i_*. Class *j* is rejected when it displays both low resolution and weak correlation and accordingly satisfies the Mahalanobis distance criteria $\frac{g_{j}-\mu_{g}}{\sigma_{g}}<-1.5$ (or *res_j_* > 30 Å; default or user provided) and $\frac{{\mathrm{cc}}_{j}-\mu_{\mathrm{cc}}}{\sigma_{\mathrm{cc}}}<-1.5$. De-selected classes typically have resolutions >15–20 Å, subject to data set-dependent variations.

With the K3 camera, collection rates have escalated and refining parameters of a continuously expanding dataset routinely scaling to several millions of particles has become computationally challenging. Therefore, we developed for the 2D stream analysis an incremental learning update strategy similar to that used in our 3D refinement approach and cisTEM (Grant et al., 2018, Reboul et al., 2018b). Hence, only a fraction δ of randomly selected particles of the global set are subjected to refinement. Initially, δ is set to 40% but is adaptively lowered as the global set expands such that 500 K particles are always refined against the current global set of classes. Empirically we have found that this strategy efficiently improves the class averages vs. the previous iterations while delivering substantial speedups when dealing very large (>10^7^) particle sets.

### 2.6 Generation of an ab initio 3D model and preparation of data for 3D operations

**Fig. 4:**
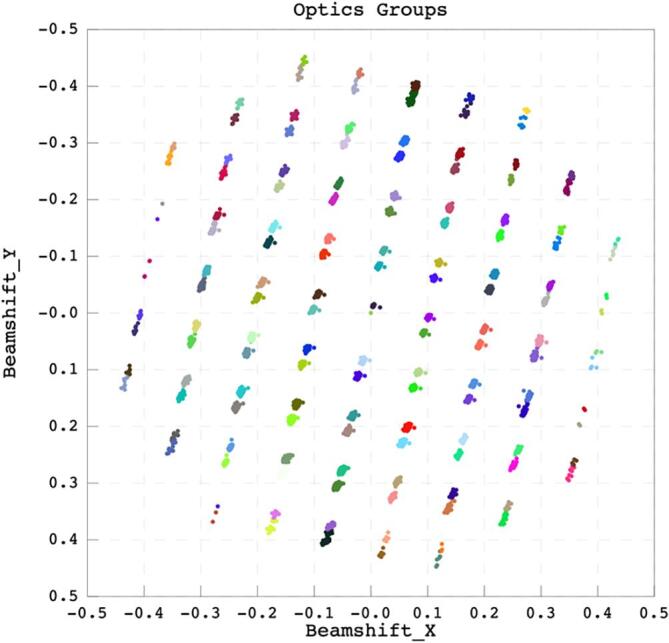
Optics Group Assignment. Plot of beam shift in × and y for 7,428 movies collected using EPU colored by optics group assignment. Hierarchical clustering is used to group movies based on beam shift, before each group is further divided into sub-populations based on the location identifier in the EPU filename. The user may limit the maximum population of each group and/or apply an offset to the optics group number to aid dataset combination. The data shown were collected using a 1.2/1.3 quantifoil grid with two shots per hole using AFIS beam shift collection in EPU 2.7 (Thermo Fisher Scientific, The Netherlands).

As previously described, the approach taken in SIMPLE 3.0 for *ab initio* model generation is based on use of the signal-enhanced 2D class averages rather than the noisier particles (Reboul et al., 2016, Reboul et al., 2018a). This significantly reduces the computational load as a few hundred class averages need to be oriented rather than many thousands of particles. Once an *ab intio* model is generated the user then continues to process the data through 3D classification and refinement in the software of their choosing; e.g. stay within the SIMPLE 3.0 package or use alternates such as RELION 3.1 (Zivanov et al., 2020), cryoSPARC (Punjani et al., 2017) among others. The latter stages implemented in SIMPLE 3.0 will not be discussed further as they have been previously described (Elmlund et al., 2013, Reboul et al., 2016, Reboul et al., 2018a, Reboul et al., 2019, Reboul et al., 2018b). Export routines are also provided to directly generate star files and directory structures to facilitate downstream processing in RELION 3.1 (Zivanov et al., 2020) or other SPA packages, including automatic assignment of optics groups for RELION3.1 (Zivanov et al., 2020) based on beam shifts and tilts reported by EPU sessions (Fig. 4). This is done by hierarchical clustering of movies based on beam-shift coordinates output by EPU into .xml files followed by division of clusters into sub-populations based on the location identifiers encoded in the EPU file name. This allows accurate separation of movies derived from different beam tilts used for multiple shots per hole as well as separation from holes collected by beam shifts.

### 2.7 Example use case—the structure of stenotrophomonas maltophilia methylcrontonoyl-CoA carboxylase (MCC)

**Fig. 5:**
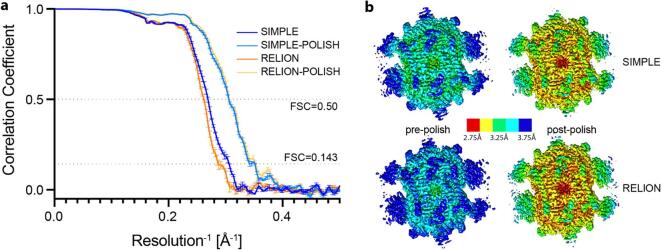
FSC curves and Local Resolution colored volumes for the same particle (and half set assignments) processed with either SIMPLE3.0 or RELION 3.1. (a) Blue curves are for data where patched motion and CTF estimation was performed in SIMPLE3.0 before (dark blue) and after (light blue) CTF refinement and Bayesian Polishing in RELION 3.1. Orange curves are for the same particles extracted from RELION 3.1 motion corrected and CTFFIND 4 CTF estimated movies before (dark orange) and after (light orange) CTF refinement and Bayesian Polishing in RELION 3.1. All volumes were refined in three independent calculations and the values shown are the mean +/- SD of the FSC values obtained. (b) Example volumes from each protocol are shown colored by local resolution (calculated in RELION 3.1). (For interpretation of the references to colour in this figure legend, the reader is referred to the web version of this article.)

MCC from *Stenotrophomonas maltophilia* was purified, cryo grids prepared and data collected as described in Supplementary Materials, S3. Our streaming pipeline without picking was used to motion correct/CTF estimate an initial chunk of 20 movies. These were hand-picked within the SIMPLE 3.0 GUI, particles extracted, 2D clustering performed and selected class averages used as picking references for a restarted streaming analysis. The level of secondary structure visible and the variety of view distributions seen in the streaming 2D analysis suggested that the data would be suitable for high resolution structure determination, so data collection was continued for ∼18 h resulting in 1853 movies. At the end of data collection, selected 2D classes were used to generate an *ab intio* 3D model and the data were exported to RELION 3.1 (Zivanov et al., 2020) for rounds of 3D classification, 3D autorefinement, CTFrefinement and Bayesian polishing to generate a volume with resolution 2.8 Å (gold standard FSC 0.143 criterion). Having identified the subset of particles which yielded a high-resolution volume we next re-processed the data in a variety of ways, then re-extracted the same particles maintaining the same validation half-sets to allow comparison between the SIMPLE3.0 motion correction and the corresponding routines implemented in RELION 3.1, *i.e.* reimplementation of MotionCorr and CTFFIND 4. Fig. 5 shows the FSC curves and local resolution colored volumes derived from the common particle sets both before and after further CTFrefinement and Bayesian polishing.

Using SIMPLE 3.0 to do anisotropic motion correction and CTF estimation yields a higher resolution volume prior to further CTF refinement/Bayesian polishing compared to that generated entirely within RELION 3.1 at the same stage. Once CTF refinement and polishing have been performed, both processing workflows yield volumes of the same quality. The improvement obtained by the more advanced anisotropic corrections implemented in SIMPLE3.0 at an early stage of 3D refinement may be critical for data sets that lie on the success/failure boundary of the current methodology. Furthermore, the algorithms in SIMPLE 3.0 have been heavily optimized to keep up with K3 data collection using only minimal CPU computing resources.

## 3 Conclusions

The protocols, algorithms, data organization and visualization tools included in SIMPLE 3.0 have been generally applied for the majority of the samples imaged in our facility in Oxford. The computationally lightweight approach in SIMPLE 3.0 has enabled rapid identification of samples unlikely to generate 3D volumes of sufficient resolution to address the biological question at hand through rapid screening of samples at the level of the 2D class averages. Applied across many samples, SIMPLE 3.0 has also helped identify potentially useful samples and accelerated the rate of high-resolution 3D structure determination by SPA.

## Declaration of Competing Interest

The authors declare that they have no known competing financial interests or personal relationships that could have appeared to influence the work reported in this paper.
