# Supplementary material for: SIMPLE 3.0. Stream single-particle cryo-EM analysis in real time
Source: J Struct Biol X. 2020 Nov 7;4:100040. doi: 10.1016/j.yjsbx.2020.100040 (PMC7695977; doi:10.1016/j.yjsbx.2020.100040)
Supplement: Supplementary data 1 [file mmc1.pdf]

Supplementary Material

S.1. Stage drift and beam-induced motion correction

S.1.1 Movie frames alignment

For a given set of frames F^n, where $n = 1, \dots, N_F$, the objective is to find a set of shifts that best describe the stage drift and beam-induced motion for each frame. This set of shifts $\left\{\left(s_x, s_y\right)_n\right\}$ is determined such that the sum of cross-correlations between shifted frames $F\left(s_x, s_y\right)^n$ and their respective references R^n is maximized. For a given frame, the corresponding reference is a weighted sum of all frames expect for itself, *i.e.*

$$R^n \equiv \sum_{m \neq n} w_m F(x, y)^m$$

The weights $\sum_m w_m = 1$ are chosen as detailed below (S.1.3). For frames $\hat{F}^n$ and references $\hat{R}^n$ expressed in Fourier representation, the cross-correlation is defined as

$$cc\left(\hat{F}^n, \hat{R}^n\right) \equiv \frac{\sum_{hk} \hat{F}^n_{hk} \cdot \left(\hat{R}^n_{hk}\right)^*}{\sqrt{\sum_{hk}\left|\hat{F}^n_{hk}\right|^2 \cdot \sum_{hk}\left|\hat{R}^n_{hk}\right|^2}},$$

where h, k denote the Fourier pixel indices and the summation is carried out over an appropriately chosen resolution range. In order to incorporate a B-factor into the calculation of the cross-correlation, the frames are multiplied by a factor of $\exp\left(-\frac{b}{4}\left(\frac{h}{N_x} + \frac{k}{N_y}\right)^2\right)$ where b is a user-specified constant and N_x/ N_y are the number of pixels along the x/y dimensions. The determination of shifts (alignment) is performed in two stages. In the first stage, a coarse search with a subsequent sub-pixel precision is performed (S.1.2). This is followed by a gradient-based continuous optimization (S.1.4).

S.1.2 Coarse search

The images are Fourier-transformed and a resolution range is selected. For every frame n, the matrix

$$P^n \equiv \frac{\mathcal{FT}^{-1}\{\hat{F}^n.(\hat{R}^n)^*\}}{\sqrt{\sum_{hk}\left|\hat{F}^n_{hk}\right|^2.\sum_{hk}\left|\hat{R}^n_{hk}\right|^2}}$$

is calculated, where $\mathcal{FT}^{-1}$ indicates the inverse Fourier transform. Within a reasonable search window around the centre of P^n, the index i_{max}, j_{max} denotes the best whole-pixel shift. Subpixel shifts are determined by considering the values of P^n adjacent to i_{max}, j_{max} and approximating P^n at these values including i_{max}, j_{max} through of a quadratic function. The position of the maximum of this quadratic function denotes the subpixel shift. It is found via

$$i_{max}^{sub} = \frac{\alpha_x - \gamma_x}{\alpha_x + \gamma_x - 2\beta}$$

$$j_{max}^{sub} = \frac{\alpha_y - \gamma_y}{\alpha_y + \gamma_y - 2\beta}$$

where $\beta \equiv P(i_{max} + 1, j_{max})$, $\alpha_x \equiv P(i_{max} + 1, j_{max})$, $\gamma_x \equiv P(i_{max} - 1, j_{max})$ and $\alpha_y \equiv P(i_{max}, j_{max} + 1)$, $\gamma_y \equiv P(i_{max}, j_{max} - 1)$. The identified shifts are applied to the frames and the weights calculated. The new references are calculated using the new weights and shifts and the procedure outlined above is repeated iteratively until convergence. In Fourier representation, the shifts can be applied to the frame F^n via the operation

$$\hat{F}^n(s_x, s_y)_{hk} = \hat{F}^n{}_{hk} \,.\, e^{2\pi i\left(\frac{s_x.h}{N_x} + \frac{s_y.k}{N_y}\right)} \,.$$

S.1.3 Calculation of frame weights

Given a set of correlations $\{c_1, \ldots, c_n\}$, the ratio between maximum and minimum value $\Delta \equiv \mathrm{c_{max}}/\mathrm{c_{min}}$ is determined. If Δ is greater than $\Delta_{thres} = 1.5$, the correlations are normalized via

$$c_i \leftarrow \frac{e^{\frac{c_i - c_{min}}{\Delta}} - 1}{e - 1}.$$

This decreases the variation between correlations, thus resulting in a smoother weight distribution. The weights from the correlations are then obtained through a softmax-formula via

$$w_i = \frac{e^{-(1-c_i)}}{\sum_j e^{-(1-c_j)}}.$$

The weights are chosen to be uniform initially.

S.1.4 Continuous method

The references are calculated using the shifts and weights determined in the previous phase (coarse search). For each frame n, the cross-correlation with its reference is considered and a continuous L-BFGS-B optimizer is applied to determine a potentially better shift. The objective function is given by

$$cc\left(\hat{F}^n(s_x, s_y), \hat{R}^n\right) \equiv \frac{\sum_{hk} \hat{F}^n_{hk}(s_x, s_y) \,.\, \left(\hat{R}^n_{hk}\right)^*}{\sqrt{\sum_{hk}\left|\hat{F}^n_{hk}\right|^2 . \sum_{hk}\left|\hat{R}^n_{hk}\right|^2}},$$

With the definition of a shifted frame in Fourier representation, one finds the gradient via

$$\frac{\delta}{\delta s_x} cc\left(\hat{F}^n(s_x, s_y), \hat{R}^n\right) \equiv \frac{2\pi i \,.\, s_x}{N_x} \frac{\sum_{hk} k \,.\, \hat{F}^n_{hk}(s_x, s_y) \,.\, \left(\hat{R}^n_{hk}\right)^*}{\sqrt{\sum_{hk}\left|\hat{F}^n_{hk}\right|^2 . \sum_{hk}\left|\hat{R}^n_{hk}\right|^2}},$$

$$\frac{\delta}{\delta s_y} cc\left(\hat{F}^n(s_x, s_y), \hat{R}^n\right) \equiv \frac{2\pi i. s_y}{N_y} \frac{\sum_{hk} l \,.\, \hat{F}^n_{hk}(s_x, s_y) \,.\, \left(\hat{R}^n_{hk}\right)^*}{\sqrt{\sum_{hk}\left|\hat{F}^n_{hk}\right|^2 . \sum_{hk}\left|\hat{R}^n_{hk}\right|^2}},$$

The optimized shifts determined are applied, new references are calculated and the procedure outlined above is repeated iteratively. Here, the weights are kept constant.

S.2. Analytical gradients for CTF parameters estimation

We aim at finding the gradients of the objective function f with respect to the defoci Δf_x and Δf_y (*cf.* Material & Methods for details of notations and abbreviations):

$$f = cc + f_{pen} \qquad \text{(S2.1)}$$

with:

$$cc = \frac{\sum_{\boldsymbol{g}} F(\boldsymbol{g})|CTF(\boldsymbol{g})|}{\sqrt{\sum_{\boldsymbol{g}} F(\boldsymbol{g})^2 \sum_{\boldsymbol{g}} CTF(\boldsymbol{g})^2}},$$

$$f_{pen} = -\frac{1}{2N}\left(\frac{\Delta f_x - \Delta f_y}{\Delta\Delta f_{tol}}\right)^2$$

$$CTF(\boldsymbol{g}) = -\sin\left[\pi\lambda\boldsymbol{g}^2\left(\Delta f - \frac{1}{2}\lambda^2\boldsymbol{g}^2 C_s\right) + \Delta\phi + A\right]$$

and $\Delta f = \frac{1}{2}\left[\Delta f_x + \Delta f_y + (\Delta f_x - \Delta f_y)\cos(2[\alpha(\boldsymbol{g}) - \alpha_a])\right]$

We first seek to derive f with respect to Δf_x:

$$\frac{\partial f}{\partial \Delta f_x} = \frac{\partial cc}{\partial \Delta f_x} + \frac{\partial f_{pen}}{\partial \Delta f_x} \qquad \text{(S2.2)}$$

Focusing first on the cross-correlation between experimental and theoretical CTF, F is centred and standardized such that $\sum_{\boldsymbol{g}} F(\boldsymbol{g}) = 0$, $\sum_{\boldsymbol{g}} F(\boldsymbol{g})^2 = N$ and cc (Eq. S2.1) simplifies to:

$$cc = \frac{\sum_{\boldsymbol{g}} F(\boldsymbol{g})|CTF(\boldsymbol{g})|}{\sqrt{N\sum_{\boldsymbol{g}} CTF(\boldsymbol{g})^2}}.$$

Using the chain-rule we find:

$$\frac{\partial cc}{\partial \Delta f_x} = \frac{\partial cc}{\partial \Delta f}\frac{\partial \Delta f}{\partial \Delta f_x} \qquad \text{(S2.3)}$$

It can then be shown that:

$$\frac{\partial cc}{\partial \Delta f} = \frac{\sum_{\boldsymbol{g}} F(\boldsymbol{g})\frac{\partial |CTF(\boldsymbol{g})|}{\partial \Delta f}}{\sqrt{N\sum_{\boldsymbol{g}} CTF(\boldsymbol{g})^2}} - N\frac{\sum_{\boldsymbol{g}} F(\boldsymbol{g})|CTF(\boldsymbol{g})|}{\left(N\sum_{\boldsymbol{g}} CTF(\boldsymbol{g})^2\right)^{3/2}}\sum_{\boldsymbol{g}} CTF(\boldsymbol{g})\frac{\partial CTF(\boldsymbol{g})}{\partial \Delta f}$$

where

$$\frac{\partial|CTF(\boldsymbol{g})|}{\partial\Delta f}=\frac{\partial CTF(\boldsymbol{g})}{\partial\Delta f}\frac{CTF(\boldsymbol{g})}{|CTF(\boldsymbol{g})|},$$

$$\frac{\partial CTF(\boldsymbol{g})}{\partial\Delta f}=-\pi\lambda\boldsymbol{g}^2\cos\left[\pi\lambda\boldsymbol{g}^2\left(\Delta f-\frac{1}{2}\lambda^2\boldsymbol{g}^2C_s\right)+\Delta\phi+A\right],$$

$$\frac{\partial\Delta f}{\partial\Delta f_x}=\frac{1}{2}[1+\cos(2[\alpha(\boldsymbol{g})-\alpha_a])].$$

We next derive f_{pen} (right term in equation S.2.1) with respect to Δf_x :

$$\frac{\partial f_{pen}}{\partial\Delta f_x}=-\frac{\Delta f_x-\Delta f_y}{N\Delta\Delta {f_{tol}}^2}$$

Consequently, all the terms necessary for evaluating the gradient of f with respect to Δf_x have been determined. We may trivially derive the gradient with respect to Δf_y by substituting Δf_x with Δf_y in equation S.2.2 and S.2.3 and obtain $\frac{\partial f}{\partial\Delta f_y}$ noting that

$\frac{\partial\Delta f}{\partial\Delta f_y}=1-\frac{\partial\Delta f}{\partial\Delta f_x}$ and $\frac{\partial f_{pen}}{\partial\Delta f_y}=-\frac{\partial f_{pen}}{\partial\Delta f_x}$.

S.3. Material and methods

S.3.1 Purification of Methylcrotonyl-CoA carboxylase (MCC) from *Stenotrophomonas maltophilia*

Methylcrotonyl-CoA carboxylase (MCC) from *Stenotrophomonas maltophilia* was purified from a membrane preparation of contaminated *E. coli* culture that had been grown in terrific broth supplemented with 0.1 % (w/v) rhamnose monohydrate and 50 µg/ml kanamycin. Briefly, cells from the mixed culture were resuspended in TBS (100 mM Tris, 150 mM NaCl, 1 mM EDTA pH 8.0) containing 30 µg/mL DNase I and 400 µg/mL lysozyme for 30 mins before passage through an EmulsiFlex C5 homogenizer (Avestin) at 15,000 psi. Following removal of unbroken cells by centrifugation at 24,000*g* for 20 min, membranes were collected by centrifugation at 200,000*g* for 1.5 h and solubilized in TBS containing 1% (w/v) lauryl

maltose neopentyl glycol (LMNG) for 2 h at 4°C. Insoluble material was then removed by centrifugation at 100,000*g* for 30 min. Solubilized membranes were applied to a Streptactin XT column. The resin was washed with 10 column volumes (CV) of TBS containing 0.02% (w/v) LMNG and proteins were eluted in 5 CV of TBS supplemented with 0.02% (w/v) LMNG and 50 mM D-biotin. Eluates were concentrated using a 100-kDa molecular weight cutoff (MWCO) centrifugal filter unit and injected onto a Superose 6 Increase 10/300 GL size exclusion column pre-equilibrated in TBS plus 0.02% (w/v) LMNG. Peak fractions were collected and concentrated using a 100-kDa MWCO centrifugal filter unit.

S.3.2 Cryo grid preparation and data acquisition

Four microliters of purified MCC (A_{280nm} = 0.77) was adsorbed for 10 s onto glow-discharged Quantifoil grids (300 mesh, Au R1.2/1.3) followed by blotting for 2 s (100% humidity, 8°C) and plunge frozen in liquid ethane using a Vitrobot Mark IV. Data were collected in counting mode on a Titan Krios G3 operating at 300 kV with a GIF energy filter and K2 Summit detector using a calibrated pixel size of 0.822 Å, a dose rate of 4.05 e^-/pix/s, and an exposure of 8 s, corresponding to a total dose of 48 $e^-/Å^2$. Movies (1,845) were fractionated over 32 frames and collected for 18 h using EPU.

Supplementary Figure 1

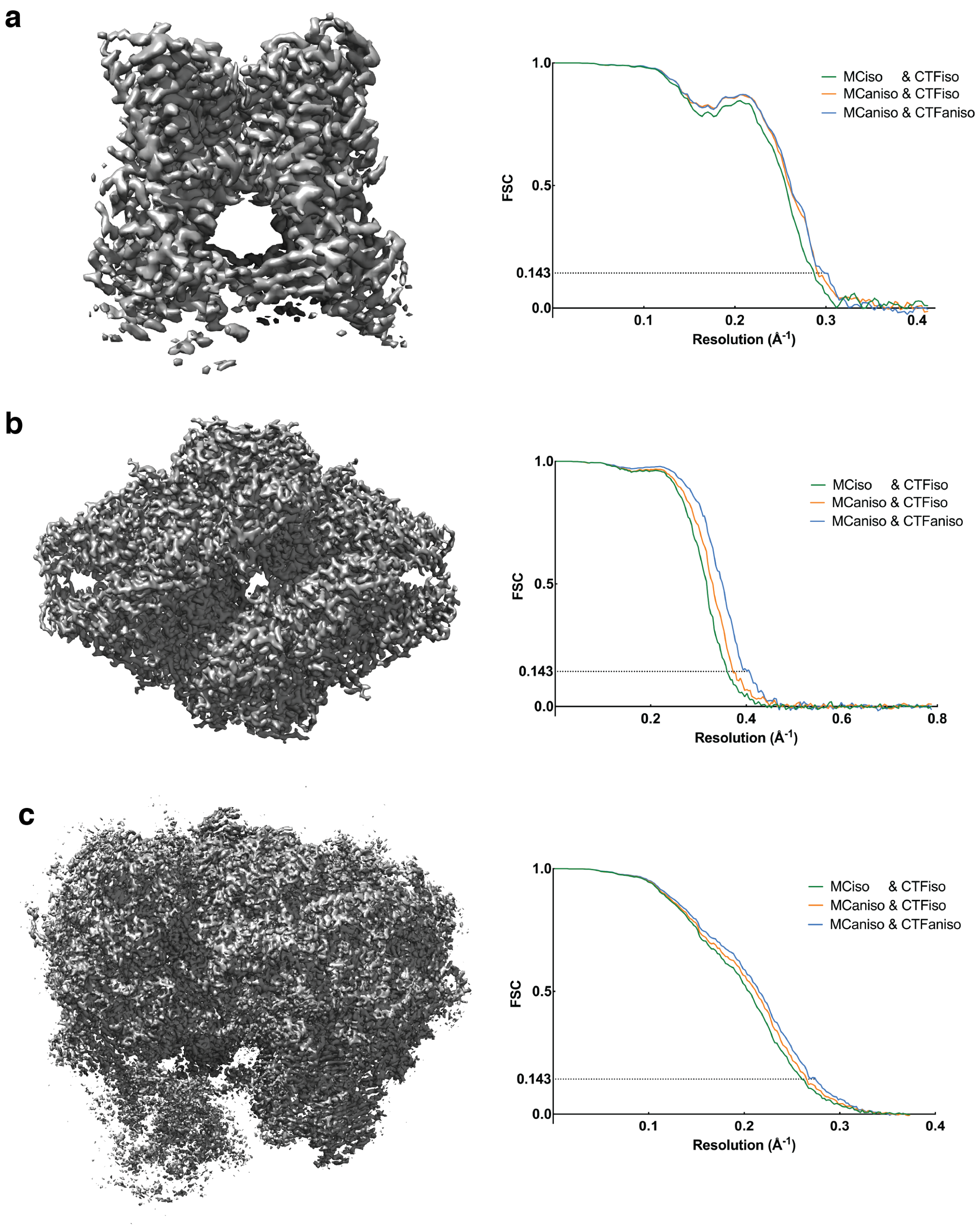

Validation of the proposed motion correction and CTF parameters estimation methods.

For each dataset, three different algorithms were tested: 1) MCiso+CTFiso, isotropic motion correction followed by isotropic CTF parameters estimation; 2) MCaniso+CTFiso, isotropic &

anisotropic motion correction followed by CTFiso; and 3) MCaniso+CTFaniso: MCaniso followed by our patch CTF method (CTFaniso). Particles extraction employed the publicly deposited particles coordinates and thus yielded one stack per algorithm. After 2D classification and *ab initio* 3D model generation, each set of particles was individually refined using r*efine3D* in SIMPLE3.0 with gold-standard resolution estimation and spherical masking. a) TPRV1 (EMPIAR10005). Left panel: 3D model corresponding to the MCaniso+CTFaniso algorithm and displaying near-atomic resolution features. FSC curves are presented on the right panel. The resolutions obtained (FSC=0.143) are for the three algorithms 3.50, 3.46 and 3.35Å respectively. b) β-galactosidase (EMPIAR10061). The resolutions obtained are 2.78, 2.70 and 2.47Å. c) Ribosome 80S (EMPIAR10028). The resolutions obtained are 3.86, 3.77 and 3.65Å.
